# 4-[(Ethoxy­imino)(phen­yl)meth­yl]-5-methyl-2-phenyl-1*H*-pyrazol-3(2*H*)-one

**DOI:** 10.1107/S1600536808026263

**Published:** 2008-08-20

**Authors:** Jiu-Si Wang, Yan-Ling Jiang, Wen-Kui Dong, Li Xu, Ai-Ping Kong

**Affiliations:** a School of Chemical and Biological Engineering, Lanzhou Jiaotong University, Lanzhou 730070, People’s Republic of China

## Abstract

In the mol­ecule of the title compound, C_19_H_19_N_3_O_2_, the central pyrazole ring makes dihedral angles of 9.89 (3) and 66.06 (5)° with the two phenyl rings, and the two phenyl rings form an angle of 74.05 (5)°. An intra­molecular C—H⋯O hydrogen bond forms a six-membered ring, producing an *S*(6) ring motif. In the crystal structure, inter­molecular N—H⋯O and C—H⋯O hydrogen bonds link each mol­ecule to two others, forming an infinite one-dimensional supra­molecular structure along the *c* axis.

## Related literature

For related literature, see: Beeam *et al.* (1984 ▶); Bonati (1980 ▶); Dong & Feng (2006 ▶); Dong *et al.* (2008*a* ▶,*b* ▶); Duan *et al.* (2007 ▶).
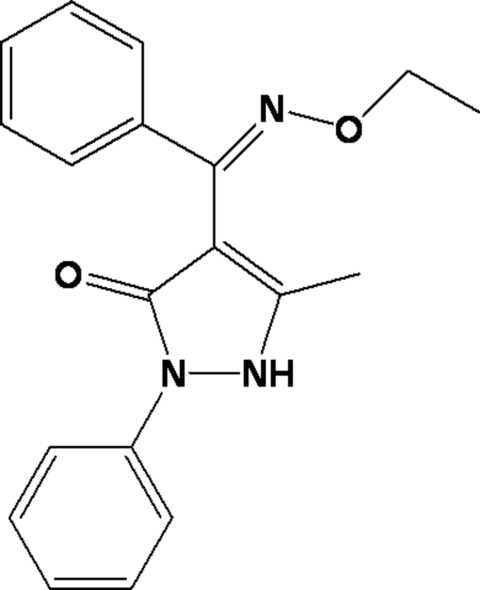


## Experimental

### 

#### Crystal data

C_19_H_19_N_3_O_2_
*M* *_r_* = 321.37
Monoclinic, 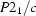

*a* = 13.0046 (15) Å
*b* = 11.4657 (11) Å
*c* = 11.6874 (12) Å
β = 99.4530 (10)°
*V* = 1719.0 (3) Å^3^
*Z* = 4
Mo *K*α radiation
μ = 0.08 mm^−1^
*T* = 298 (2) K
0.50 × 0.18 × 0.16 mm

#### Data collection

Brucker SMART 1000 CCD area-detector diffractometer
Absorption correction: multi-scan (*SADABS*; Sheldrick, 1996 ▶) *T*_min_ = 0.960, *T*_max_ = 0.987
8480 measured reflections
3028 independent reflections
1756 reflections with *I* > 2σ(*I*)
*R*_int_ = 0.041

#### Refinement

*R*[*F* ^2^ > 2σ(*F* ^2^)] = 0.044
*wR*(*F* ^2^) = 0.140
*S* = 1.02
3028 reflections
219 parameters
H-atom parameters constrained
Δρ_max_ = 0.17 e Å^−3^
Δρ_min_ = −0.23 e Å^−3^

### 

Data collection: *SMART* (Siemens, 1996 ▶); cell refinement: *SAINT* (Siemens, 1996 ▶); data reduction: *SAINT*; program(s) used to solve structure: *SHELXS97* (Sheldrick, 2008 ▶); program(s) used to refine structure: *SHELXL97* (Sheldrick, 2008 ▶); molecular graphics: *SHELXTL* (Sheldrick, 2008 ▶); software used to prepare material for publication: *SHELXTL*.

## Supplementary Material

Crystal structure: contains datablocks global, I. DOI: 10.1107/S1600536808026263/zl2137sup1.cif

Structure factors: contains datablocks I. DOI: 10.1107/S1600536808026263/zl2137Isup2.hkl

Additional supplementary materials:  crystallographic information; 3D view; checkCIF report

## Figures and Tables

**Table 1:** Hydrogen-bond geometry (Å, °)

*D*—H⋯*A*	*D*—H	H⋯*A*	*D*⋯*A*	*D*—H⋯*A*
N3—H3⋯O2^i^	0.86	1.80	2.653 (2)	171
C15—H15⋯O2^i^	0.93	2.42	3.200 (3)	141
C19—H19⋯O2	0.93	2.32	2.900 (3)	120

Symmetry code: (i) 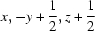.

**Fig. 1.**
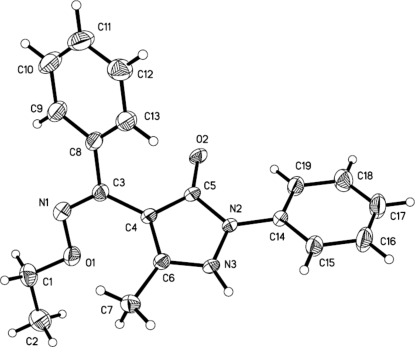
ORTEP representation of the title compound with atom numbering. Displacement ellipsoids for non-hydrogen atoms are drawn at the 30% probability level.

**Fig. 2.**
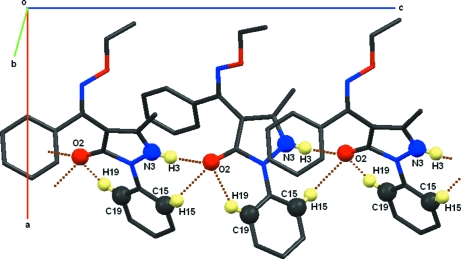
Part of the supramolecular structure of the title compound viewed along the b axis. Intra- and intermolecular hydrogen bonds are shown as dashed lines.

## supplementary crystallographic information

### Comment

The pyrazole ring is a prominent structural motif found in numerous pharmaceutically active compounds. Due to the easy preparation and rich biological activity, the pyrazole framework plays an essential role in biologically active compounds and therefore represents an interesting template for combinatorial as well as medicinal chemistry (Beeam *et al.*, 1984; Bonati *et al.*, 1980). As an extension of our work (Dong *et al.*, 2006; Duan *et al.*, 2007; Dong *et al.*, 2008*a*; Dong *et al.*, 2008*b*) on the structural characterization of oxime compounds, the title compound, (Fig. 1), is reported here.

The single-crystal structure of the title compound is built up by only the C_19_H_19_N_3_O_2_ molecules, in which all bond lengths are in normal ranges. In the title compound, the central pyrazole ring makes dihedral angles of 9.89 (3) and 66.06 (5)° with the two outer benzene rings, and the two outer benzene rings form an angle of 74.05 (5)°. An intramolecular C—H···O hydrogen bond forms a six-membered ring, producing an S(6) ring motif. In the crystal structure, intermolecular N—H···O and C—H···O hydrogen bonds link each molecule to two others, forming an infinite one-dimensional supramolecular structure along the *c* axis (Fig. 2).

### Experimental

To a solution of 1-phenyl-3-methyl-4-benzoyl-5-pyrazolon (5 mmol) in warm ethanol (5 ml) was added an ethanol (5 ml) solution of ethoxyamine (10 mmol). After stirring the reaction mixture at 338 K for 6 h, the solvent was removed under reduced pressure and the residue was recrystallized from ethanol to give the title compound. Yield, 68%. mp. 444–445 K. Anal. Calc. for C_19_H_19_N_3_O_2_: C, 71.01; H, 5.96; N, 13.08. Found: C, 71.32; H, 5.81; N, 13.15.

Colorless prismatic crystals suitable for single-crystal X-ray diffraction were obtained by recrystallization from ethanol at room temperature.

### Refinement

Non-H atoms were refined anisotropically. H atoms were treated as riding atoms with distances C—H = 0.96 (CH_3_), C—H = 0.97 (CH_2_), or 0.93 Å (CH), N—H = 0.86 Å, and *U*_iso_(H) = 1.2 *U*_eq_(C) and 1.5 *U*_eq_(O).

### Crystal data

**Table:**

C_19_H_19_N_3_O_2_	*F*_000_ = 680
*M**_r_* = 321.37	*D*_x_ = 1.242 Mg m^−^^3^
Monoclinic, *P*2_1_/*c*	Mo *K*α radiation λ = 0.71073 Å
Hall symbol: -P 2ybc	Cell parameters from 1770 reflections
*a* = 13.0046 (15) Å	θ = 2.4–22.5º
*b* = 11.4657 (11) Å	µ = 0.08 mm^−^^1^
*c* = 11.6874 (12) Å	*T* = 298 (2) K
β = 99.4530 (10)º	Prismatic, colorless
*V* = 1719.0 (3) Å^3^	0.50 × 0.18 × 0.16 mm
*Z* = 4	

### Data collection

**Table:**

Brucker SMART 1000 CCD area-detector diffractometer	3028 independent reflections
Radiation source: fine-focus sealed tube	1756 reflections with *I* > 2σ(*I*)
Monochromator: graphite	*R*_int_ = 0.041
*T* = 298(2) K	θ_max_ = 25.0º
φ and ω scans	θ_min_ = 1.6º
Absorption correction: multi-scan(SADABS; Sheldrick, 1996)	*h* = −15→15
*T*_min_ = 0.960, *T*_max_ = 0.987	*k* = −13→9
8480 measured reflections	*l* = −13→13

### Refinement

**Table:**

Refinement on *F*^2^	Secondary atom site location: difference Fourier map
Least-squares matrix: full	Hydrogen site location: inferred from neighbouring sites
*R*[*F*^2^ > 2σ(*F*^2^)] = 0.044	H-atom parameters constrained
*wR*(*F*^2^) = 0.140	*w* = 1/[σ^2^(*F*_o_^2^) + (0.0687*P*)^2^ + 0.0591*P*] where *P* = (*F*_o_^2^ + 2*F*_c_^2^)/3
*S* = 1.02	(Δ/σ)_max_ < 0.001
3028 reflections	Δρ_max_ = 0.17 e Å^−^^3^
219 parameters	Δρ_min_ = −0.23 e Å^−^^3^
Primary atom site location: structure-invariant direct methods	Extinction correction: none

### Special details

**Table:**

Geometry. All e.s.d.'s (except the e.s.d. in the dihedral angle between two l.s. planes) are estimated using the full covariance matrix. The cell e.s.d.'s are taken into account individually in the estimation of e.s.d.'s in distances, angles and torsion angles; correlations between e.s.d.'s in cell parameters are only used when they are defined by crystal symmetry. An approximate (isotropic) treatment of cell e.s.d.'s is used for estimating e.s.d.'s involving l.s. planes.
Refinement. Refinement of *F*^2^ against ALL reflections. The weighted *R*-factor *wR* and goodness of fit *S* are based on *F*^2^, conventional *R*-factors *R* are based on *F*, with *F* set to zero for negative *F*^2^. The threshold expression of *F*^2^ > σ(*F*^2^) is used only for calculating *R*-factors(gt) *etc*. and is not relevant to the choice of reflections for refinement. *R*-factors based on *F*^2^ are statistically about twice as large as those based on *F*, and *R*- factors based on ALL data will be even larger.

### Fractional atomic coordinates and isotropic or equivalent isotropic displacement parameters (Å^2^)

**Table:**

	*x*	*y*	*z*	*U*_iso_*/*U*_eq_	
N1	0.53222 (14)	0.34824 (17)	−0.06367 (17)	0.0486 (5)	
N2	0.84502 (13)	0.23475 (16)	0.16076 (15)	0.0369 (5)	
N3	0.78882 (13)	0.29323 (15)	0.23325 (15)	0.0386 (5)	
H3	0.8064	0.2996	0.3072	0.046*	
O1	0.49758 (11)	0.28139 (15)	0.02320 (14)	0.0546 (5)	
O2	0.82634 (12)	0.20296 (15)	−0.03673 (13)	0.0529 (5)	
C1	0.38665 (18)	0.2834 (2)	0.0039 (3)	0.0623 (8)	
H1A	0.3595	0.2505	−0.0715	0.075*	
H1B	0.3617	0.3630	0.0061	0.075*	
C2	0.3515 (2)	0.2130 (3)	0.0975 (3)	0.0805 (10)	
H2A	0.3776	0.1349	0.0954	0.121*	
H2B	0.2767	0.2113	0.0859	0.121*	
H2C	0.3774	0.2474	0.1715	0.121*	
C3	0.63196 (17)	0.35862 (19)	−0.04638 (19)	0.0395 (6)	
C4	0.70377 (15)	0.31546 (19)	0.05486 (18)	0.0363 (5)	
C5	0.79385 (17)	0.24753 (19)	0.04860 (19)	0.0371 (5)	
C6	0.70309 (16)	0.33834 (19)	0.17079 (19)	0.0360 (5)	
C7	0.62708 (17)	0.4013 (2)	0.2301 (2)	0.0496 (7)	
H7A	0.5735	0.3484	0.2446	0.074*	
H7B	0.5964	0.4640	0.1817	0.074*	
H7C	0.6622	0.4323	0.3023	0.074*	
C8	0.67571 (18)	0.4228 (2)	−0.1379 (2)	0.0432 (6)	
C9	0.6212 (2)	0.4282 (2)	−0.2505 (2)	0.0576 (7)	
H9	0.5572	0.3908	−0.2688	0.069*	
C10	0.6611 (3)	0.4881 (3)	−0.3347 (3)	0.0755 (9)	
H10	0.6238	0.4914	−0.4097	0.091*	
C11	0.7555 (3)	0.5432 (3)	−0.3093 (3)	0.0773 (9)	
H11	0.7823	0.5832	−0.3671	0.093*	
C12	0.8107 (2)	0.5397 (2)	−0.1988 (3)	0.0664 (8)	
H12	0.8743	0.5782	−0.1811	0.080*	
C13	0.77084 (19)	0.4782 (2)	−0.1138 (2)	0.0516 (7)	
H13	0.8089	0.4743	−0.0393	0.062*	
C14	0.94564 (15)	0.19033 (19)	0.20265 (19)	0.0358 (5)	
C15	0.99788 (18)	0.2239 (2)	0.3093 (2)	0.0517 (7)	
H15	0.9678	0.2775	0.3535	0.062*	
C16	1.09499 (19)	0.1782 (3)	0.3508 (2)	0.0659 (8)	
H16	1.1296	0.2005	0.4235	0.079*	
C17	1.1406 (2)	0.1007 (3)	0.2862 (3)	0.0667 (8)	
H17	1.2063	0.0706	0.3143	0.080*	
C18	1.0891 (2)	0.0678 (2)	0.1801 (3)	0.0647 (8)	
H18	1.1203	0.0153	0.1358	0.078*	
C19	0.99121 (17)	0.1113 (2)	0.1374 (2)	0.0520 (7)	
H19	0.9564	0.0875	0.0652	0.062*	

### Atomic displacement parameters (Å^2^)

**Table:**

	*U*^11^	*U*^22^	*U*^33^	*U*^12^	*U*^13^	*U*^23^
N1	0.0485 (12)	0.0550 (13)	0.0405 (13)	0.0035 (10)	0.0019 (10)	0.0055 (10)
N2	0.0349 (10)	0.0546 (12)	0.0209 (10)	0.0067 (8)	0.0036 (8)	0.0001 (9)
N3	0.0394 (10)	0.0560 (12)	0.0200 (10)	0.0020 (9)	0.0038 (8)	−0.0052 (9)
O1	0.0391 (9)	0.0719 (12)	0.0507 (11)	−0.0002 (8)	0.0010 (8)	0.0123 (9)
O2	0.0585 (10)	0.0778 (12)	0.0223 (9)	0.0200 (9)	0.0069 (8)	−0.0005 (8)
C1	0.0402 (14)	0.0705 (19)	0.073 (2)	0.0002 (13)	−0.0002 (14)	0.0023 (16)
C2	0.0532 (17)	0.109 (3)	0.080 (2)	−0.0073 (16)	0.0143 (16)	0.007 (2)
C3	0.0398 (13)	0.0446 (14)	0.0328 (13)	0.0031 (10)	0.0020 (10)	−0.0023 (11)
C4	0.0359 (12)	0.0465 (13)	0.0258 (13)	−0.0004 (10)	0.0034 (10)	0.0039 (10)
C5	0.0396 (12)	0.0490 (14)	0.0225 (12)	0.0010 (10)	0.0039 (10)	0.0016 (11)
C6	0.0354 (12)	0.0423 (13)	0.0297 (13)	−0.0027 (10)	0.0036 (10)	0.0004 (10)
C7	0.0479 (14)	0.0618 (16)	0.0400 (15)	0.0050 (12)	0.0098 (12)	−0.0069 (13)
C8	0.0518 (14)	0.0436 (14)	0.0332 (14)	0.0124 (12)	0.0038 (11)	0.0021 (11)
C9	0.0661 (17)	0.0619 (17)	0.0428 (17)	0.0110 (14)	0.0026 (14)	0.0081 (14)
C10	0.096 (2)	0.084 (2)	0.0456 (19)	0.0188 (19)	0.0093 (17)	0.0216 (17)
C11	0.101 (3)	0.076 (2)	0.062 (2)	0.0172 (19)	0.034 (2)	0.0259 (17)
C12	0.0723 (19)	0.0598 (18)	0.072 (2)	0.0014 (15)	0.0254 (17)	0.0119 (16)
C13	0.0570 (16)	0.0532 (16)	0.0447 (16)	0.0036 (13)	0.0088 (13)	0.0033 (13)
C14	0.0325 (12)	0.0446 (13)	0.0293 (13)	0.0011 (10)	0.0025 (10)	0.0048 (11)
C15	0.0437 (14)	0.0661 (17)	0.0423 (16)	0.0060 (12)	−0.0023 (12)	−0.0078 (13)
C16	0.0507 (16)	0.086 (2)	0.0528 (18)	0.0087 (15)	−0.0162 (14)	−0.0103 (16)
C17	0.0473 (15)	0.080 (2)	0.066 (2)	0.0174 (15)	−0.0093 (15)	0.0000 (17)
C18	0.0568 (16)	0.0708 (19)	0.065 (2)	0.0203 (14)	0.0056 (15)	−0.0057 (16)
C19	0.0487 (14)	0.0649 (17)	0.0395 (15)	0.0124 (13)	−0.0011 (12)	−0.0048 (13)

### Geometric parameters (Å, °)

**Table:**

N1—C3	1.285 (3)	C8—C13	1.378 (3)
N1—O1	1.404 (2)	C8—C9	1.389 (3)
N2—C5	1.377 (3)	C9—C10	1.371 (4)
N2—N3	1.380 (2)	C9—H9	0.9300
N2—C14	1.415 (3)	C10—C11	1.369 (4)
N3—C6	1.333 (3)	C10—H10	0.9300
N3—H3	0.8600	C11—C12	1.371 (4)
O1—C1	1.423 (3)	C11—H11	0.9300
O2—C5	1.254 (2)	C12—C13	1.386 (3)
C1—C2	1.491 (4)	C12—H12	0.9300
C1—H1A	0.9700	C13—H13	0.9300
C1—H1B	0.9700	C14—C15	1.373 (3)
C2—H2A	0.9600	C14—C19	1.379 (3)
C2—H2B	0.9600	C15—C16	1.380 (3)
C2—H2C	0.9600	C15—H15	0.9300
C3—C4	1.468 (3)	C16—C17	1.363 (3)
C3—C8	1.487 (3)	C16—H16	0.9300
C4—C6	1.382 (3)	C17—C18	1.362 (4)
C4—C5	1.419 (3)	C17—H17	0.9300
C6—C7	1.485 (3)	C18—C19	1.383 (3)
C7—H7A	0.9600	C18—H18	0.9300
C7—H7B	0.9600	C19—H19	0.9300
C7—H7C	0.9600		
			
C3—N1—O1	111.86 (18)	H7B—C7—H7C	109.5
C5—N2—N3	108.11 (16)	C13—C8—C9	118.3 (2)
C5—N2—C14	130.00 (18)	C13—C8—C3	121.2 (2)
N3—N2—C14	121.03 (17)	C9—C8—C3	120.5 (2)
C6—N3—N2	109.39 (17)	C10—C9—C8	120.5 (3)
C6—N3—H3	125.3	C10—C9—H9	119.7
N2—N3—H3	125.3	C8—C9—H9	119.7
N1—O1—C1	108.28 (17)	C11—C10—C9	120.5 (3)
O1—C1—C2	107.5 (2)	C11—C10—H10	119.7
O1—C1—H1A	110.2	C9—C10—H10	119.7
C2—C1—H1A	110.2	C10—C11—C12	120.2 (3)
O1—C1—H1B	110.2	C10—C11—H11	119.9
C2—C1—H1B	110.2	C12—C11—H11	119.9
H1A—C1—H1B	108.5	C11—C12—C13	119.4 (3)
C1—C2—H2A	109.5	C11—C12—H12	120.3
C1—C2—H2B	109.5	C13—C12—H12	120.3
H2A—C2—H2B	109.5	C8—C13—C12	121.1 (3)
C1—C2—H2C	109.5	C8—C13—H13	119.5
H2A—C2—H2C	109.5	C12—C13—H13	119.5
H2B—C2—H2C	109.5	C15—C14—C19	119.5 (2)
N1—C3—C4	126.0 (2)	C15—C14—N2	120.3 (2)
N1—C3—C8	115.4 (2)	C19—C14—N2	120.1 (2)
C4—C3—C8	118.62 (19)	C14—C15—C16	120.0 (2)
C6—C4—C5	107.05 (19)	C14—C15—H15	120.0
C6—C4—C3	128.4 (2)	C16—C15—H15	120.0
C5—C4—C3	124.40 (19)	C17—C16—C15	120.6 (3)
O2—C5—N2	122.71 (19)	C17—C16—H16	119.7
O2—C5—C4	130.8 (2)	C15—C16—H16	119.7
N2—C5—C4	106.46 (18)	C18—C17—C16	119.4 (2)
N3—C6—C4	108.91 (19)	C18—C17—H17	120.3
N3—C6—C7	119.62 (19)	C16—C17—H17	120.3
C4—C6—C7	131.5 (2)	C17—C18—C19	121.0 (3)
C6—C7—H7A	109.5	C17—C18—H18	119.5
C6—C7—H7B	109.5	C19—C18—H18	119.5
H7A—C7—H7B	109.5	C14—C19—C18	119.4 (2)
C6—C7—H7C	109.5	C14—C19—H19	120.3
H7A—C7—H7C	109.5	C18—C19—H19	120.3
			
C5—N2—N3—C6	2.1 (2)	N1—C3—C8—C13	155.2 (2)
C14—N2—N3—C6	172.45 (18)	C4—C3—C8—C13	−23.6 (3)
C3—N1—O1—C1	174.5 (2)	N1—C3—C8—C9	−24.8 (3)
N1—O1—C1—C2	−179.5 (2)	C4—C3—C8—C9	156.4 (2)
O1—N1—C3—C4	−4.5 (3)	C13—C8—C9—C10	−0.6 (4)
O1—N1—C3—C8	176.79 (17)	C3—C8—C9—C10	179.4 (2)
N1—C3—C4—C6	−55.2 (4)	C8—C9—C10—C11	0.3 (4)
C8—C3—C4—C6	123.5 (2)	C9—C10—C11—C12	−0.5 (4)
N1—C3—C4—C5	130.1 (2)	C10—C11—C12—C13	1.1 (4)
C8—C3—C4—C5	−51.3 (3)	C9—C8—C13—C12	1.1 (3)
N3—N2—C5—O2	−179.5 (2)	C3—C8—C13—C12	−178.9 (2)
C14—N2—C5—O2	11.3 (4)	C11—C12—C13—C8	−1.4 (4)
N3—N2—C5—C4	−0.3 (2)	C5—N2—C14—C15	155.0 (2)
C14—N2—C5—C4	−169.5 (2)	N3—N2—C14—C15	−13.0 (3)
C6—C4—C5—O2	177.7 (2)	C5—N2—C14—C19	−26.1 (3)
C3—C4—C5—O2	−6.7 (4)	N3—N2—C14—C19	165.9 (2)
C6—C4—C5—N2	−1.4 (2)	C19—C14—C15—C16	−0.4 (4)
C3—C4—C5—N2	174.25 (19)	N2—C14—C15—C16	178.5 (2)
N2—N3—C6—C4	−3.0 (2)	C14—C15—C16—C17	0.9 (4)
N2—N3—C6—C7	177.33 (17)	C15—C16—C17—C18	−0.5 (4)
C5—C4—C6—N3	2.8 (2)	C16—C17—C18—C19	−0.3 (4)
C3—C4—C6—N3	−172.7 (2)	C15—C14—C19—C18	−0.4 (4)
C5—C4—C6—C7	−177.7 (2)	N2—C14—C19—C18	−179.3 (2)
C3—C4—C6—C7	6.9 (4)	C17—C18—C19—C14	0.8 (4)

### Hydrogen-bond geometry (Å, °)

**Table:**

*D*—H···*A*	*D*—H	H···*A*	*D*···*A*	*D*—H···*A*
N3—H3···O2^i^	0.86	1.80	2.653 (2)	171
C15—H15···O2^i^	0.93	2.42	3.200 (3)	141
C19—H19···O2	0.93	2.32	2.900 (3)	120

Symmetry codes: (i) *x*, −*y*+1/2, *z*+1/2.
